# Rapid detection of pandemic influenza in the presence of seasonal influenza

**DOI:** 10.1186/1471-2458-10-726

**Published:** 2010-11-24

**Authors:** Brajendra K Singh, Nicholas J Savill, Neil M Ferguson, Chris Robertson, Mark EJ Woolhouse

**Affiliations:** 1 Centre for Infectious Diseases, University of Edinburgh, Ashworth Laboratories, King's Buildings, West Mains Road, Edinburgh, EH9 3JT, UK; 2 MRC Centre for Outbreak Analysis and Modelling, Department of Infectious Disease Epidemiology, Imperial College London, Norfolk Place, London W2 1PG, UK; 3 Department of Mathematics and Statistics, Strathclyde University, 26 Richmond Street, Glasgow G1 1XH, UK; 4 Health Protection Scotland, Clifton House, Clifton Place, Glasgow G3 7LN, UK

## Abstract

**Background:**

Key to the control of pandemic influenza are surveillance systems that raise alarms rapidly and sensitively. In addition, they must minimise false alarms during a normal influenza season. We develop a method that uses historical syndromic influenza data from the existing surveillance system 'SERVIS' (Scottish Enhanced Respiratory Virus Infection Surveillance) for influenza-like illness (ILI) in Scotland.

**Methods:**

We develop an algorithm based on the weekly case ratio (WCR) of reported ILI cases to generate an alarm for pandemic influenza. From the seasonal influenza data from 13 Scottish health boards, we estimate the joint probability distribution of the country-level WCR and the number of health boards showing synchronous increases in reported influenza cases over the previous week. Pandemic cases are sampled with various case reporting rates from simulated pandemic influenza infections and overlaid with seasonal SERVIS data from 2001 to 2007. Using this combined time series we test our method for speed of detection, sensitivity and specificity. Also, the 2008-09 SERVIS ILI cases are used for testing detection performances of the three methods with a real pandemic data.

**Results:**

We compare our method, based on our simulation study, to the moving-average Cumulative Sums (Mov-Avg Cusum) and ILI rate threshold methods and find it to be more sensitive and rapid. For 1% case reporting and detection specificity of 95%, our method is 100% sensitive and has median detection time (MDT) of 4 weeks while the Mov-Avg Cusum and ILI rate threshold methods are, respectively, 97% and 100% sensitive with MDT of 5 weeks. At 99% specificity, our method remains 100% sensitive with MDT of 5 weeks. Although the threshold method maintains its sensitivity of 100% with MDT of 5 weeks, sensitivity of Mov-Avg Cusum declines to 92% with increased MDT of 6 weeks. For a two-fold decrease in the case reporting rate (0.5%) and 99% specificity, the WCR and threshold methods, respectively, have MDT of 5 and 6 weeks with both having sensitivity close to 100% while the Mov-Avg Cusum method can only manage sensitivity of 77% with MDT of 6 weeks. However, the WCR and Mov-Avg Cusum methods outperform the ILI threshold method by 1 week in retrospective detection of the 2009 pandemic in Scotland.

**Conclusions:**

While computationally and statistically simple to implement, the WCR algorithm is capable of raising alarms, rapidly and sensitively, for influenza pandemics against a background of seasonal influenza. Although the algorithm was developed using the SERVIS data, it has the capacity to be used at other geographic scales and for different disease systems where buying some early extra time is critical.

## Background

Rapid detection of pandemic influenza at national or regional level is a public health issue of critical importance [1,2]. Huge excess mortality and morbidity have been associated with the pandemics of influenza outbreaks in the past [3]. In the aftermath of the highly pathogenic H5N1 avian influenza outbreaks worldwide [4,5], the growing concern [3,4] of a virulent form of a possible human influenza pandemic has led to the setting up of influenza surveillance systems across the globe [6]. One of the main purposes of such worldwide expansion of influenza surveillance systems is the timely detection of influenza outbreaks of pandemic potential [7]. The importance of timely detection lies in buying some extra time for being prepared to deal with a pandemic [3,8,9]. This has also been corroborated by some recent mathematical modelling studies [10,11] of pandemic influenza outbreaks: a key finding suggests that there would be a small window of opportunity for getting ahead of pandemic outbreak fronts and thus helping early pandemic mitigation efforts if it could be detected early on.

Most developed countries as well as many from the developing world have some form of influenza surveillance in place [6]. These surveillance systems are based on the reporting of disease syndromes (e.g., reports of Influenza-like illnesses (ILI)) and are generally designed to monitor levels of seasonal influenza [12,13]. Although the signature of pandemic influenza could be different from that of seasonal ones [14], the traditional approach (patients presenting with clinical signs of ILI, collection of throat/nasal swab samples from some of these patients and, finally, laboratory confirmation of influenza) followed in influenza surveillance systems, in the absence of any detection algorithm applied to syndromic data, may not be able to pick it up early on. This is the reason that public health surveillance systems are being supplemented by the new state-of-the-art statistical tools [1,2]. The development of these new statistical tools has demonstrated the potential to automate syndromic surveillance systems, to be able to raise specific and sensitive early alerts of adverse disease outbreaks. Indeed this is a fast growing and a very active area of scientific research at the moment [6].

At present, a number of methods [12,13,15] exist to establish the onset of peak activities in the epidemics of seasonal influenza. These methods are mostly based on regression [16,17] or time-series [12,13,15] analysis of seasonal ILI data. One such method is the Moving-Average Cumulative sums (Mov-Avg Cusum) method [18-20]. Originally developed for the industrial quality control [21], it is now frequently used for detecting the outbreaks of seasonal and pandemic influenza [12,22]. Recently there has been a flurry of new detection methods based on sophisticated statistical approaches [1,2], including those aimed at real-time monitoring and projecting of influenza cases [23,24]. However, challenges remain in terms of how to use the ILI surveillance data in a simple and efficient manner for timely detection of influenza pandemics.

The basic reproduction ratio R_0 _(i.e., the average number of new infections produced by a single infection in a totally naïve host population) plays a central role in our understanding of infectious disease dynamics. It determines whether a new infection will successfully invade the susceptible population [25,26]. In the case of an ongoing epidemic, the effective reproduction ratio R replaces R_0 _[25]. In the presence of disease tracing data, R can be estimated and the in- or out-of-control status of an ongoing epidemic can be established [27]. Where there is no availability of disease tracing data, as in influenza syndromic data, the weekly case ratio (WCR), defined as the ratio of the number of reported cases in a week to the number of cases reported in the previous week, may function as an indirect measure of R and may be suitable for raising public health alarms in the early stages of an emerging infection. Although pandemic influenza infections may grow exponentially in early invading stages (evident in the mortality data from the past pandemics [28] or in the mathematical modelling [10,11,29] of influenza pandemic), the detection algorithms so far employed in influenza surveillance systems largely ignore this natural behaviour as the basis for generating early warning of influenza outbreaks of pandemic potential.

The aim of this paper is to develop a detection algorithm, based on the estimates of WCR for expected influenza pandemics, to facilitate sensitive, specific and rapid detection of a pandemic outbreak at a regional level based on existing surveillance systems. Using the influenza surveillance data from Scotland, we first sift through the spatiotemporal patterns in the historical data by calculating WCR and N_HB_, the number of health boards (HBs) that show increases in the weekly ILI cases. The joint probability distribution of WCR and N_HB _is then contrasted with expected and observed patterns in the presence of pandemic influenza. As described in the next section, the expected patterns for pandemic cases are obtained from a previously published mathematical model [29]. Observed patterns for pandemic cases are based on records from the 2008-09 season when influenza A(H1N1)v was circulating in Scotland.

We compare the performance of our detection algorithm, using simulated influenza pandemics as well as data from the 2009 influenza A(H1N1v) epidemic in Scotland, with that of the Mov-Avg Cusum method and with the ILI rate threshold method, a slightly modified form of the baseline ILI activity indicator used by the Health Protection Agency (HPA) in the monitoring of seasonal influenza in the UK.

## Methods

### Seasonal ILI data from SERVIS

In developing our pandemic detection algorithm, we used historical seasonal ILI data, collected and compiled under the Scottish Enhanced Respiratory Virus Infection Surveillance (SERVIS) system. The data set spans from the 2001-02 season through to the 2008-09 season. Seasonal ILI cases are normally reported weekly over a period of 33 weeks (from the first week of October to the third week of May) in different age- and sex-classes, by sentinel general practices (GPs) across Scotland. The SERVIS sentinel GPs are drawn from 13 Scottish health boards. (There are currently 14 HBs in Scotland; all HBs except the Western Isles HB have participated in the SERVIS network of the sentinel GPs.) The Scottish health boards widely vary in their population sizes from 20,000 to 1,360,000. The total number of the sentinel GPs varied from 20 to 44 across the influenza seasons considered, with a minimum of 1 GP in a HB to a maximum of 9 GPs in a HB. Altogether, the numbers of people registered with sentinel GPs represent 2.7% to 4.9% of the Scottish population of around 5.15 million. This system was designed as a national surveillance scheme with regional coverage. It was not designed to be used as a surveillance system in each health board separately.

**Figure 1:**
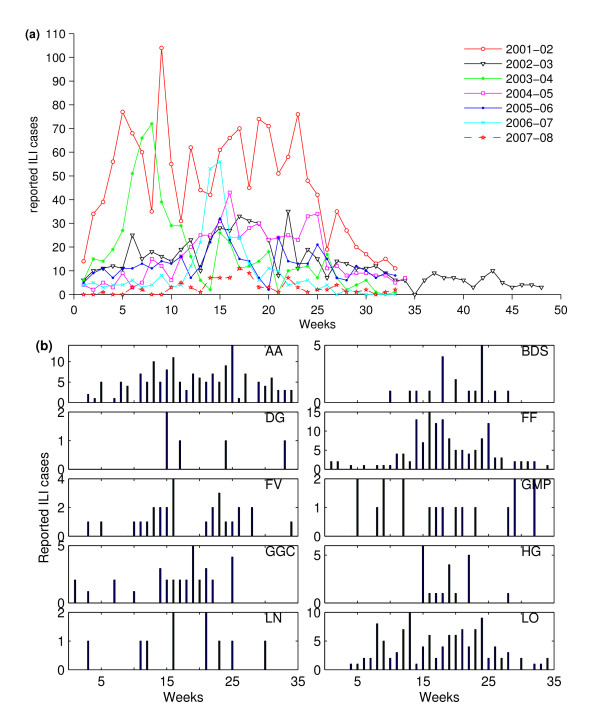
**Historical SERVIS ILI data**. (a) Weekly ILI cases reported by the SERVIS sentinel GPs aggregated at the national level. Week 1 is the first week of October, and week 33 is the third week of May. In the 2002-03 season, the influenza surveillance was continued for another 15 weeks because of the SARS epidemic. (b) The weekly reported ILI cases of the influenza season 2004-05 aggregated at the health board level. (Health boards are represented by the following letter codes. AA: Ayrshire & Arran; BDS: Borders; DG: Dumfries & Galloway; FF: Fife; FV: Forth Valley; GMP: Grampian; GGC: Greater Glasgow & Clyde; HG: Highland; LN: Lanarkshire; LO: Lothian; OR: Orkney; SH: Shetland; and TA: Tayside). In that season, only 10 out of 13 participating HBs had one or more sentinel GPs that reported ILI cases.

The weekly reported ILI cases at the national level are shown in Figure 1a. In our analysis we used the weekly ILI cases, aggregated at the HB level. An example of weekly HB-level ILI cases is shown for the 2004-05 influenza season in Figure 1b. Sentinel GPs are often recorded as reporting zero cases in a week in the SERVIS data. It is unknown whether this represents true zeroes or non-reporting. However, we believe that certainly, in the data for the 2008-09 season, a blank means that no report was made by the practice and a zero means a report was made but no cases occurred.

The historical influenza data from 6 influenza seasons from 2001-02 through 2006-07 were used in estimating the background pattern of the seasonal ILI cases. The ILI data for the season 2007-08 from 23 sentinel GPs recorded just 93 cases compared with over 300 cases for the other seasons. The whole of the UK reported influenza cases below the HPA baseline activity threshold in this season [30]. We therefore excluded the 2007-08 data from our analysis. (As we checked this and will be clear later, the inclusion of the historical ILI data of this season increases the detection efficiency of the WCR method. The exclusion of the data of this season, therefore, ensures conservative estimate for the WCR method in the performance testing.) The 2008-09 data-set contains the 2009 influenza A(H1N1)v pandemic cases, so it was used for performance testing of our detection algorithm with real pandemic data. The SERVIS ILI data used in the study is freely available from Health Protection Scotland on request (NSS.hpsflu@nhs.net) to anyone wishing to use them for any non-commercial research purposes.

### Simulated pandemic ILI cases

For our main analysis, we use simulated pandemic influenza data for Scotland. In brief, the pandemic model [29] is a stochastic, spatially structured, individual-based discrete time simulation. For the analyses carried out in this paper, the model pandemic outbreaks were run with a basic reproduction rate R_0 _of 1.7 and a generation time of 3 days. (The R_0 _value used here is slightly higher than what has been reported from various analyses [31] of the 2008-09 influenza A(H1N1)v outbreak data. But we have used a range of pandemic case reporting rates as discussed below.) Full model details are given in the Supplementary Information of [10,29].

The pandemic model was simulated 10 times for the whole of Great Britain, starting on day 1 seeded with a single infection at a randomly chosen location. In our analysis, however, we define the pandemic start week as the week when the first influenza infections occur in Scotland, which may or may not be reported. From the simulated pandemic infections, we sample ILI cases at case reporting rate of α as if they would have been reported by sentinel GPs to SERVIS (see Additional file 1). In this work we have used three values of α: 0.5, 1 and 5%. The sampled sentinel pandemic cases were then converted to the HB-level daily reported influenza cases by summing across all participating sentinel GPs of the HBs, which in turn were converted into weekly reported ILI cases to match the temporal resolution of the SERVIS data. The first wave of pandemic influenza cases could occur in the presence of seasonal cases at any time of the year. To take this into account, we add the simulated pandemic ILI cases to the seasonal ILI cases from each of the six seasons and use the resultant ILI time series for detecting pandemic, sliding the pandemic start week across the entire influenza season. We present our results using 300 (30 samples *times *10 simulated pandemics) sampled time series of pandemic cases. Here we use 10 simulated pandemics each sampled 30 times. (Note that the results are invariant with other sampling schemes, e.g., 300 time series, each sampled from 300 different simulated pandemics. The sampling is carried out after the first Scottish cases are reported, during the exponential growth phase of the epidemic. All simulated pandemics are therefore observed to have very similar temporal dynamics.) Each of these 300 time series were, in turn, overlaid with seasonal ILI time series from each of the 6 seasons, making a total of 1800 time series to be analysed for 33 (from week 1 to week 33) pandemic start weeks in a typical influenza season.

### Pandemic detection algorithm

#### Joint probability distribution of (WCR, N_HB_)

Our pandemic detection algorithm uses two metrics obtained from weekly reported ILI cases: the weekly case ratio (*WCR*) for cases aggregated across the region and N_HB_, the number of health boards reporting increases in the cases over the previous week.

The weekly *WCR *is defined as

$$WCR=\frac{\text{total ILI cases reported to all SERVIS sentinel GPs in week w}}{\text{total ILI cases reported to all SERVIS sentinel GPs in week w}-1}$$

**Figure 2:**
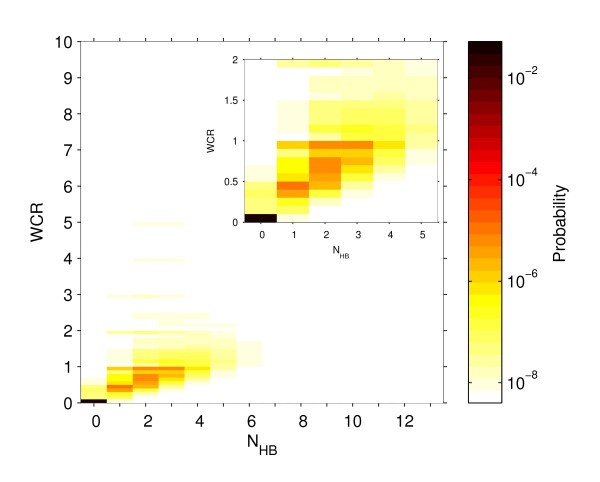
**The joint probability distribution of (WCR, N_HB_)**. The joint probability distribution of weekly case ratio (WCR), calculated from seasonal ILI cases aggregated over Scotland, and the number of health boards (N_HB_) reporting increases in the ILI cases over the previous week. This probability diagram was constructed using SERVIS data from six seasons, 2001-02 to 2006-07. WCR is binned with bin size 0.1. The inset plot zooms in on the left corner of the main plot.

Note that *WCR *is not defined for week 1 and, also, not defined for any week where in the previous week all sentinel GPs did report zero ILI cases. (In the historical data we have one instance (out of the total 214 weeks from all the 6 seasons) that for a week, which was not a start week but well within the influenza season, there were no ILI cases reported. In this situation we simply replace zero in the denominator by 1.) Since *WCR *is a continuous variable, in order to create a joint distribution, it is binned with a bin size of 0.1. We then construct a joint probability distribution of the two metrics using the historical ILI data from all the 6 seasons (see Figure 2).

### Smoothing of the joint probability

The historical seasonal ILI data are temporally heterogeneous, with substantial week-to-week variability (Figure 1). For making any useful and robust statistical inference the probability distribution requires smoothing. The smoothing was done assuming that the weekly reported ILI cases in a HB have a Poisson distribution with rate parameter equal to the reported total weekly ILI cases in the HB. The Poisson distribution is preferred to a binomial distribution as the numbers of weekly reported cases are very much smaller than the total population of a health board. We simulated the Poisson model to generate weekly ILI cases at the HB level (see Additional file 2). The model-generated ILI counts from individual runs for each season were then used, as described above, to calculate the joint distribution of *WCR *and N_HB_. A set of 10,000 model runs per season's data were used in the estimation of this joint distribution. A smaller (1,000) or larger (100,000) number of runs were also tested for the robustness of the results.

#### Specificity (*Sp*) of the detection algorithm

**Figure 3:**
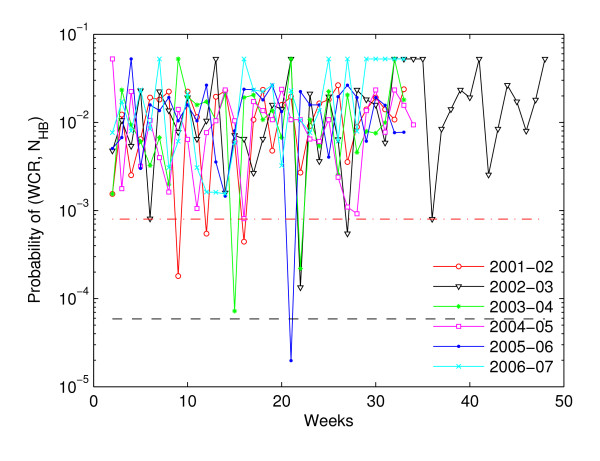
**Detection specificity of the WCR method**. An illustration of how we calculate the specificity (*Sp*) of the WCR algorithm as a function of the detection threshold δ. The straight-line plots show two values of δ: 0.000059 (dashed line) and 0.0008 (dot-dash line) which give specificity of 99% and 95% respectively. The other plots are probability values of (WCR, N_HB_) in any given week for different seasons. If the probability in any given week dips below one of the threshold probabilities, then a false positive alert for a pandemic outbreak is generated.

We tested our algorithm for its specificity (i.e., not detecting a pandemic when no pandemic is occurring). To do this, we first calculate *WCR *and N_HB _for each week from a given seasonal HB-level ILI time series. We obtain the probability values of weekly pairs of (*WCR*, N_HB_) from the joint probability distribution (the plots of the weekly probability values are given in Figure 3). In the second step, for a chosen threshold probability value δ, we count the number of weeks in which the probability of (*WCR*, N_HB_) falls below the threshold. The total counts represent the number of false alarms (*Fa_s_*(*δ*)) in season *s*. The theoretical maximum number of false alarms ($FA_{s}^{\max}$) in any given season is the total weeks minus 1 because WCR cannot be calculated for the first week. Finally, this leads us to calculate the specificity of the detection algorithm for a given threshold probability as follows:

1−(∑s=seasonsFAs(δ)∑s=seasonsFAsmax).

A detection threshold is defined as the threshold probability δ that gives rise to a pre-set specificity of our detection method. We adjust δ in order to achieve specificities of 95% and 99%. This allows us to compare our method to other algorithms which also have the same constant specificity.

#### Detecting a pandemic

**Figure 4:**
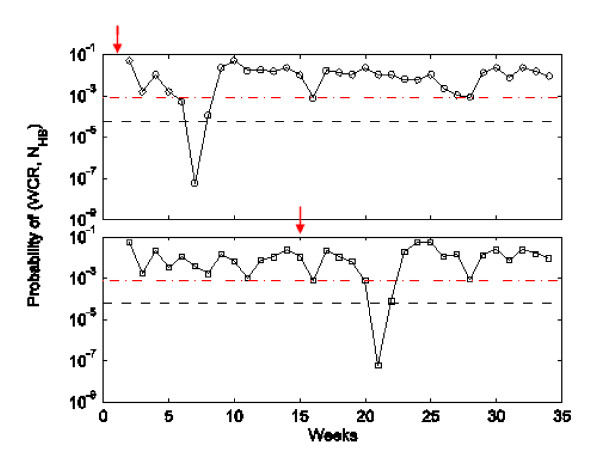
**Detection of a pandemic by the WCR method**. Probability values of weekly (WCR, N_HB_) pairs for the model pandemic cases are obtained from the distribution shown in Figure 2. Two examples, one each for pandemic starting weeks 1 (top) and 15 (bottom), are for a single run of the pandemic model. Pandemic cases were sampled at the reporting rate of 0.5% from the weekly infections and added to the 2004-05 seasonal data and WCR and N_HB _were calculated from the resultant time series. The dashed lines represent the two values of the detection threshold δ: 0.000059 (Sp = 99%) and 0.0008 (Sp = 95%). The pandemic starting on week 1 was detected on week 7, while the one starting on week 15 was detected on week 21.

Figure 4 shows an example of how our WCR detection algorithm works when applied to a simulated pandemic starting on week 1 and week 15. In the case of a pandemic starting on week 1 (week 15), the probability value of WCR and N_HB _falls below the threshold values for the first time on week 7 (week 21) triggering an alarm that week. We note here that the probability value is the probability mass function, i.e. P(*WCR *= xx and N_HB _= y), and not the more usual statistical significance level of P(*WCR *> = xx and N_HB _> = y).

### Comparison of our detection algorithm

We compared the performance (in terms of median detection time and sensitivity) of our detection algorithm with that of the Mov-Avg Cusum method. This method was recently used by Cowling et al. [12] as a statistically robust automation tool for generating early alerts for the onset of peak activity of ILI cases. We also compare the performance of an ILI rate threshold method, similar to the HPA baseline influenza activity level. The detection thresholds used by these methods were adjusted so that all three methods had the same detection specificity described earlier.

#### Moving-Average Cusum method

The *d*-week upper Cusum at time *t *is defined as follows:

$$Cusum_{t}^{+}=\max\left\{0,\frac{X_{t}-{\tilde{X}}_{t}}{{\tilde{s}}_{t}}-k+Cusum_{t-1}^{+}\right\},$$

where $Cusum_{t\leq d+7}^{+}=0$. ${\tilde{X}}_{t}$ and ${\tilde{s}}_{t}$ are the 7-week moving average and standard deviation of ILI cases in weeks *t*-*d*-1 to *t*-*d*-7 Note that *d *stands for a delay period and this method will only be informative from the (*d*+8)*^th ^*week onwards. An alarm is triggered using this method when $Cusum_{t}^{+}$ on a week *t *crosses a pre-set threshold [21]. Using the SERVIS ILI data for six seasons we preset the threshold values for this method for all 16 combinations of *d *∈{0,1,2,3}, *k *∈ {1,2} and *Sp *∈ {95%, 99%} (Figure of Additional file 3). The Mov-Avg Cusum method with *d *= 0 and *k *= 1 at *Sp *= 99% performs better than any other combination of (*d*, *k*) (Table in Additional file 3). Therefore, this combination is used to make comparisons with other methods.

#### ILI rate threshold method

The HPA set ILI consultation rates as proxies for influenza activity in the United Kingdom. The threshold rate for baseline- and epidemic-level ILI activities has recently been revised: the baseline threshold is lower from 50 to 30 consultations per 100,000 population while the epidemic threshold has been decreased from 400 to 200 consultations per 100,000 population [32]. These thresholds are derived from the time series analysis of historical seasonal data, and serve the purpose of establishing when and/or whether the community ILI activity warrants some intervention of the public health departments. In the SERVIS data set (2001 - 2007) considered, the epidemic threshold rate was never crossed [30]. Here the ILI rate threshold is denoted by η cases per 100,000 population per week and an alarm is generated when the aggregated ILI cases in any week crosses this threshold. In order to compare this method with alternatives we adjust η to obtain the pre-set specificity of 95% and 99%. The two respective values of η are 24 and 34.

## Results

### Sensitivity and median detection time

Performances of the three methods are summarised in terms of sensitivity and median detection time (MDT) in Table 1. Our algorithm is almost 100% sensitive (the lowest being 98% for a very low case reporting rate of 0.5%). Its MDT ranges from 3 to 5 weeks compared to 4 to 6 weeks for the Mov-Avg Cusum and threshold methods. While the threshold method is 100% sensitive, Mov-Avg Cusum is the worst performing method with sensitivity of 77% to 97%. Note that time to pandemic detection is counted from the start week of the first infections in simulated pandemics. There is a lag of about 3 to 5 weeks between the first infections arising in simulated pandemics and the first cases which get reported by sentinel GPs to SERVIS. If, therefore, the reference point is changed to the week of the first reported cases, then MDT of the WCR method will not be more than 2 weeks.

**Table 1:** Summary of the performances of the three methods

Case reporting rate		WCR method	Threshold method	Mov-Avg Cusum method
		Specificity = 95%		
0.5%	Sen	100	100	96
	MDT	5	5	6
1%	Sen	100	100	97
	MDT	4	5	5
5%	Sen	100	100	97
	MDT	3	4	4
		Specificity = 99%		
0.5%	Sen	98	100	77
	MDT	5	6	6
1%	Sen	100	100	92
	MDT	5	5	6
5%	Sen	100	100	95
	MDT	4	4	5

The performances of the three methods compared in terms of sensitivity (Sen) and median detection time (MDT) for different values of pandemic case reporting rates and detection specificities of 95% and 99%. Sen is given as percentage (%) of the model runs summed across all 33 weeks, i.e. calculated from a set of 1800 overlaid time series *times *33 weeks. MDT is in weeks and calculated from those of (1800 × 33) runs in which a given method was able to generate a detection alarm.

### Background seasonal ILI cases and pandemic detection times

**Figure 5:**
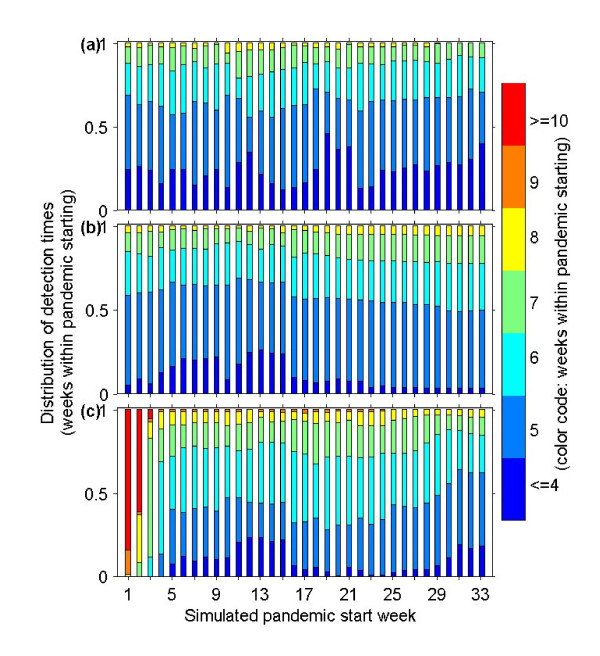
**Distribution of detection times**. A comparison between the distributions of detection times of (a) WCR algorithm, (b) ILI rate threshold, and (c) Mov-Avg Cusum method. Pandemic detection times are in terms of within ***n ***weeks of the first pandemic infections occurring; different colours of the colour bar code different values of ***n***. Individual stacked bars represent the distribution of detection times calculated for a set of 1800 overlaid time series for different pandemic start weeks, as shown on the x-axis, from week 1 to week 33 of a typical influenza season.

The distribution of detection times (Figure 5) is to show whether the temporal pattern in the background seasonal ILI cases will have any effect on pandemic detection. The detection specificity (*Sp*) and the pandemic case reporting rate (α) were set for all methods at the following values: *Sp *= 99% and α = 5%. Detection times are typically within 5-6 weeks for our method (Figure 5a). This compares favourably with the ILI rate threshold method at η = 34 cases per 100000 population (Figure 5b). The slightly longer detection times are present for the starting weeks falling in the period of late-November to mid-January (week 9 to week 15 in Figure 5a). This is the time period when the seasonal ILI incidences show *widespread *and *peak *influenza activities (Figure 1). In the case of simulated pandemics starting during this period, the above two factors together mask the probability of (*WCR*, N_HB_) as seasonal one in the first few weeks of pandemic. This masking causes delay in generating an alarm and the delay is more pronounced at lower case reporting rates and a higher specificity and is, for example, responsible for reducing the method's sensitivity to 98% at α = 0.5% and *Sp *= 99%. Conversely, during the same time-period the threshold method produces its shortest detection times because the peak-level seasonal cases having been added to the pandemic cases help the weekly ILI rate quickly cross the rate threshold η. However, our method outperforms the ILI rate threshold method in the beginning and end of an influenza season.

Mov-Avg Cusum performs poorly in terms of detection times in the first few weeks of the season (Figure 5c). This is because it requires 9 weeks to calculate the first Mov-Avg Cusum statistic. This situation improves as we move well within the seasonal period. But even for the later starting weeks, the method takes comparatively longer time to detect a pandemic. However, this method also outperforms the ILI rate threshold method towards the end of a season.

### Rapid detection

**Figure 6:**
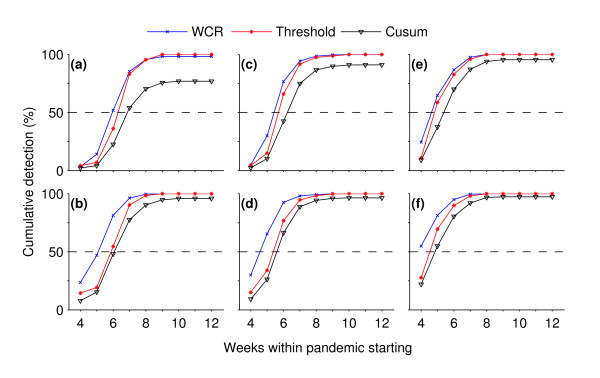
**speed of detection in the early weeks of pandemics**. Cumulative probability of detection as a function of times taken in pandemic detection (i.e., weeks within pandemic starting). These plots are for different specificity (*Sp*) and pandemic case reporting rate (α): (a) *Sp *= 99%, α = 0.5%; (b) *Sp *= 95%, α = 0.5%; (c) *Sp *= 99%, α = 1%; (d) *Sp *= 95%, α = 1%, (e) *Sp *= 99%, α = 5%; and (f) *Sp *= 95%, α = 5%. The dashed line represents the 50%-level of pandemic detections. (For each method in all subplots, the detection data were pooled together from a set of 1800 overlaid time series *times *33 weeks.)

Detection of pandemics in the early weeks of its starting depends on the case reporting rate and specificity (Figure 6). Our method outperforms the other two by rapidly detecting pandemics in a large fraction of model runs at specificity of 99% and case reporting rate α of 0.5%. It detects pandemics in >50% of total runs within the first 6 weeks of a pandemic starting while the Mov-Avg Cusum and the threshold methods detect pandemics, respectively, in <25% and <35% of total runs (Figure 6a). The time to detection decreases when the specificity was lowered from 99% to 95% (Figure 6b). In this case, about 25% and a slightly lower than 50% detection levels were achieved by the WCR method within the first 4 and 5 weeks while the Mov-Avg Cusum and the threshold methods still trailed below the 25% detection level. The same trend was observed when, for the fixed specificity of 99%, the case reporting rate α was raised from 0.5% to 1% (Figure 6c) to 5% (Figure 6e). At the elevated reporting rates, decrease in specificity further increases the detection level for all methods. But the increase in pandemic detection within the first few weeks of pandemic is more pronounced for our method than the other two (Figs 6d & 6f).

### Retrospective detection of 2009 pandemic in Scotland

**Figure 7:**
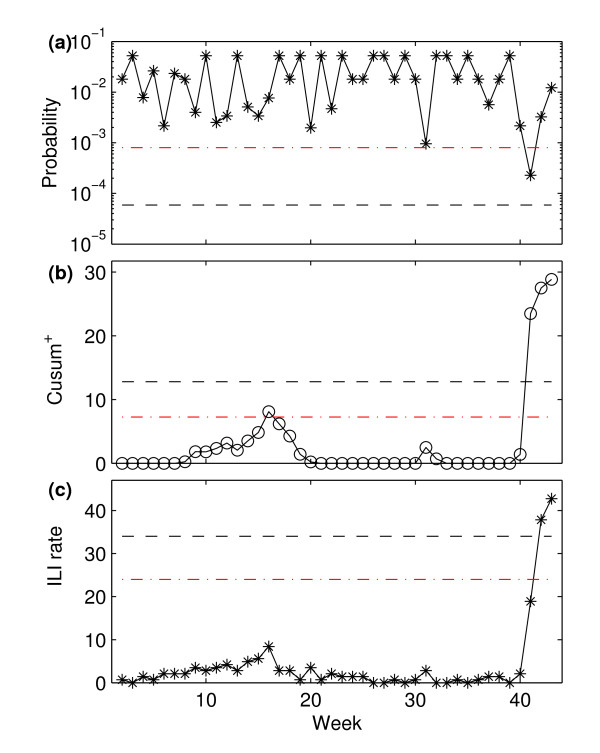
**Retrospective detection of the current pandemic using the 2009 SERVIS data**. The retrospective detection of the 2009 influenza A(H1N1)v pandemic using SERVIS ILI data. The first cases of the 2009 pandemic were reported in Scotland in the 29^th ^week of the season and our algorithm as well as the Mov-Avg Cusum method detects the pandemic 12 weeks later in week 41. The ILI threshold method detects it 1 week later in week 42.

As shown in Figure 7, our algorithm, retrospectively, detects the 2008-09 pandemic outbreak 12 weeks (i.e., on week 41) after the first cases were reported on week 29 in Scotland (Figure 7a). Clearly, here it does not perform as well as it does with the simulated pandemic data. In the next section we discuss possible reasons for this poor performance. The Mov-Avg Cusum method, which was the worst performer among the three methods using simulated pandemic data, also detects the pandemic in week 41 (Figure 7b). Both methods outperform the ILI rate threshold method by 1 week (Figure 7c).

## Discussion

In this paper we compare three methods of detecting an influenza pandemic using an existing surveillance system in Scotland called SERVIS. The ILI rate threshold method uses current ILI case data to detect pandemics. This method is motivated by the current HPA's threshold levels [30,32] to monitor influenza activity at the national scale. The HPA thresholds serve the purpose of establishing whether seasonal influenza activity warrants some intervention (e.g., the start of antiviral prescription) of the public health departments. Mov-Avg Cusum and other variants of Cusum are already being used in public health surveillance systems [1,6,12,18]. The Mov-Avg Cusum method detects a pandemic when the cumulative number of current ILI cases is substantially higher than the expected cumulative number. The Mov-Avg Cusum statistic keeps accumulating the deviation between observed and expected values over time and when the accumulated value crosses a pre-set threshold, an alarm is triggered [21]. It has three adjustable parameters that require optimisation for specific surveillance systems. Finally, the WCR algorithm introduced in this paper is based upon a characteristic of epidemics, their exponential growth in the early stages before control measures and depletion of susceptibles have occurred. It also assumes that pandemic influenza would occur synchronously across spatial units of influenza surveillance system in a region (as is predicted by the mathematical models [29] for pandemic influenza in Scotland). It makes use of the joint probability distribution derived from the historical seasonal ILI data to detect a pandemic influenza. The other methods do not use any information from the data, other than to set thresholds to achieve required specificities.

The WCR algorithm appears to provide a slightly more rapid and sensitive tool for detecting of pandemic influenza - median detection time for this method ranges from 3 to 5 weeks in comparison to 4 to 6 weeks for the other two methods. Although the WCR algorithm seems to do the job more efficiently with the simulated pandemics, it performed poorly with the 2008-09 pandemic data from SERVIS. There could be several possible reasons for this poor performance. First, the 2009 influenza A(H1N1)v epidemic happened outside the normal influenza season and, second, it was mild in severity [33,34]. In addition, the number of SERVIS sentinel GPs in the season 2008-09 was at its sparsest level - only 20 practices, as the SERVIS system was in the process of being phased out to be replaced by a system which automatically collected data from GP systems on a daily basis. These three factors will have contributed to poor reporting of the early pandemic cases, notwithstanding the huge media coverage given to the pandemic. This is consistent with the patchiness in the reported ILI cases through SERVIS sentinel GPs between weeks 29 and 41 (Additional file 4). No method will detect a pandemic in its early weeks if the early syndromic influenza data are not reported to the surveillance system. Finally, the 2009 pandemic influenza cases were more spatially heterogeneous than those predicted by the pandemic model. (It is interesting to note that during the period April to July 2009, when there was a sentinel practice within an outbreak area, Greenock and Govanhill in the GGC HB, there was no increased reporting of ILI.) This might have contributed to the observed patchiness in the sentinel reporting.

An important aspect of our algorithm is that the detection threshold remains constant throughout. An implementation of a time-varying detection threshold could make this algorithm capable of using the seasonal ILI pattern more efficiently. In principle, this could be implemented by calculating the joint probability of (WCR, N_HB_) either on a week-by-week basis, or on a slightly more coarse temporal scale of the time-windows of high/low ILI activities in the seasonal data. Clearly increasing the number of sentinel GPs and the frequency of ILI case reporting would improve the temporal resolution of the WCR algorithm. In future work we will explore how many sentinel GPs are required to achieve this aim.

Furthermore, in outbreaks of a novel influenza strain, generally children and young adults of the population, who will have little or no prior immunity to the disease [4], are disproportionately affected [28]. Implementing our detection algorithm using these data attributes will further improve the timeliness and specificity of the detection of pandemic influenza. SERVIS data contain age attributes which could be incorporated into our algorithm, but this requires more sentinel GPs to be of use.

The WCR algorithm could be applied to any syndromic surveillance data structured by space and time. Syndromic data-sets include, but are not limited to, the triage nurse calls [35] (e.g., the NHS24/NHSdirect calls in the UK [36-38]), the over-the-counter medicine sales data [39,40] available in most of the developed countries, or online web search queries [41]. These data sets are highly useful in the early detection of unusual health events [1,2]. Generally these data sets come with spatiotemporal attributes and, therefore, could potentially be integrated with the seasonal ILI data; this should enhance the detection process (in terms of timeliness, specificity and sensitivity) of pandemic influenza.

## Conclusions

While computationally simple to implement, the WCR algorithm is capable of raising alarms, rapidly and sensitively, for influenza pandemics against a background of seasonal influenza. That it has the potential to be more specific in generating alarms for pandemic influenza could be exploited for making it more cost-effective for public health surveillance systems that collect the syndromic data at a more finer spatial and/or temporal resolution. Although the algorithm was developed using the SERVIS data, it has the capacity to be used at other geographic scales and for different disease systems where buying some early extra time is critical.

More generally, we suggest that a combination of different statistical methods should be employed in generating alarms for infectious disease outbreaks. If carefully implemented, this would provide two benefits: 1) increased sensitivity; 2) different detection methods would provide cross-checks on one another, boosting confidence in the outputs of the surveillance system as a whole.

## Competing interests

The authors declare that they have no competing interests.

## Authors' contributions

BKS conceived the idea of the WCR algorithm, wrote the computer codes, ran the analysis and drafted the manuscript. MEJW and NJS originally conceived the idea of developing a pandemic detection method using the existing Scottish syndromic surveillance systems, helped with the development of the WCR algorithm as well as the analysis and with drafting the manuscript. NMF provided the simulated pandemic influenza data for Scotland. CR suggested the comparison with the Mov-Avg Cusum method, helped with the analysis and provided statistical support in the development of WCR algorithm, and helped in getting access to SERVIS data. All authors have read and approved the final manuscript.

## Pre-publication history

The pre-publication history for this paper can be accessed here:

http://www.biomedcentral.com/1471-2458/10/726/prepub

## Supplementary Material

Additional file 1 **Details of the sampling of reported pandemic ILI cases**. This details how the simulated pandemic cases were sampled at the GP level from the model-generated daily influenza infections at the postcode district level. Click here for file

Additional file 2 **Smoothing of seasonal ILI data**. This figure shows the outputs of the Poisson model for all the six influenza seasons from 2001-02 to 2006-07. Click here for file

Additional file 3 **Detection thresholds for different (*d*, *k*) pairs and the selection of the best of the Mov-Avg Cusum (*d, k*) method**. This describes the finding of the detection threshold values of different Mov-Avg Cusum models and the selection of the best among them to make a comparison with the WCR method. Click here for file

Additional file 4 **Seasonal SERVIS ILI data for the influenza season 2008-09**. This figure presents the weekly time series of seasonal SERVIS ILI cases at the HB level for the season 2008-09. Click here for file

## References

[B1] Lawson A Kleinman K Spatial and syndromatic surveillance for public health 2005 Chichester: Wiley

[B2] Lombardo JS Buckeridge DL Disease surveillance: a public health informatics approach 2007 Chichester: John Wiley

[B3] WHO Avian influenza: assessing the pandemic threat 2005 World Health Organization

[B4] Monto AS The threat of an avian influenza pandemic N Engl J Med 2005 352 323 325 10.1056/NEJMp048343 15668220

[B5] Monto AS Comanor L Shay DK Thompson WW Epidemiology of pandemic influenza: use of surveillance and modeling for pandemic preparedness J Infect Dis 2006 194 Suppl 2 S92 97 10.1086/507559 17163395

[B6] Yan P Chen H Zeng D Syndromic Surveillance Systems Annual Review of Information Science and Technology 2008 42 425 495 10.1002/aris.2008.1440420117

[B7] Sebastiani P Mandl KD Szolovits P Kohane IS Ramoni MF A Bayesian dynamic model for influenza surveillance Stat Med 2006 25 1803 1816 discussion 1817-1825 10.1002/sim.2566 16645996 PMC4128871

[B8] Gensheimer KF Fukuda K Brammer L Cox N Patriarca PA Strikas RA Preparing for pandemic influenza: the need for enhanced surveillance Emerg Infect Dis 1999 5 297 299 10.3201/eid0502.990219 10221887 PMC2640704

[B9] WHO Influenza pandemic preparedness plan: the role of WHO and guidelines for national and regional planning 1999 [Geneva]: WHO, Dept. of Communicable Disease Surveillance and Response

[B10] Ferguson NM Cummings DA Cauchemez S Fraser C Riley S Meeyai A Iamsirithaworn S Burke DS Strategies for containing an emerging influenza pandemic in Southeast Asia Nature 2005 437 209 214 10.1038/nature04017 16079797

[B11] Longini IM Jr Nizam A Xu S Ungchusak K Hanshaoworakul W Cummings DA Halloran ME Containing pandemic influenza at the source Science 2005 309 1083 1087 10.1126/science.1115717 16079251

[B12] Cowling BJ Wong IO Ho LM Riley S Leung GM Methods for monitoring influenza surveillance data Int J Epidemiol 2006 35 1314 1321 10.1093/ije/dyl162 16926216

[B13] Hashimoto S Murakami Y Taniguchi K Nagai M Detection of epidemics in their early stage through infectious disease surveillance Int J Epidemiol 2000 29 905 910 10.1093/ije/29.5.905 11034976

[B14] Michael AS A Bayesian dynamic model for influenza surveillance by Sebastiani et al Statistics in Medicine 2006 25 1817 1818 10.1002/sim.2565 PMC4128871 16645996

[B15] Farrington CP Andrews NJ Beale AD Catchpole MA A Statistical Algorithm for the Early Detection of Outbreaks of Infectious Disease Journal of the Royal Statistical Society Series A (Statistics in Society) 1996 159 547 563 10.2307/2983331

[B16] Serfling RE Methods for current statistical analysis of excess pneumonia-influenza deaths Public Health Rep 1963 78 494 506 19316455 PMC1915276

[B17] Costagliola D Flahault A Galinec D Garnerin P Menares J Valleron AJ A routine tool for detection and assessment of epidemics of influenza-like syndromes in France Am J Public Health 1991 81 97 99 10.2105/AJPH.81.1.97 1983924 PMC1404927

[B18] Rogerson PA Yamada I Approaches to syndromic surveillance when data consist of small regional counts MMWR Morb Mortal Wkly Rep 2004 53 Suppl 79 85 15714634

[B19] Hutwagner L Browne T Seeman GM Fleischauer AT Comparing aberration detection methods with simulated data Emerg Infect Dis 2005 11 314 316 15752454 10.3201/eid1102.040587 PMC3320440

[B20] Hutwagner LC Maloney EK Bean NH Slutsker L Martin SM Using laboratory-based surveillance data for prevention: an algorithm for detecting Salmonella outbreaks Emerg Infect Dis 1997 3 395 400 10.3201/eid0303.970322 9284390 PMC2627626

[B21] Montgomery DC Introduction to statistical quality control 1996 3 New York, NY; London: Wiley

[B22] Griffin B Jain A Davies-Cole J Glymph C Lum G Washington S Stoto M Early detection of influenza outbreaks using the DC Department of Health's syndromic surveillance system BMC Public Health 2009 9 483 10.1186/1471-2458-9-483 20028535 PMC2807869

[B23] Hall IM Gani R Hughes HE Leach S Real-time epidemic forecasting for pandemic influenza Epidemiol Infect 2007 135 372 385 10.1017/S0950268806007084 16928287 PMC2870596

[B24] Ong JBS Chen MIC Cook AR Lee HC Lee VJ Lin RTP Tambyah PA Goh LG Real-Time Epidemic Monitoring and Forecasting of H1N1-2009 Using Influenza-Like Illness from General Practice and Family Doctor Clinics in Singapore PLoS ONE 2010 5 e10036 10.1371/journal.pone.0010036 20418945 PMC2854682

[B25] Matthews L Woolhouse M New approaches to quantifying the spread of infection Nat Rev Microbiol 2005 3 529 536 10.1038/nrmicro1178 15995653 PMC7096817

[B26] Matthews L Woolhouse ME Hunter N The basic reproduction number for scrapie Proc Biol Sci 1999 266 1085 1090 10.1098/rspb.1999.0747 10380685 PMC1689932

[B27] Woolhouse M Chase-Topping M Haydon D Friar J Matthews L Hughes G Shaw D Wilesmith J Donaldson A Cornell S Keeling M Grenfell B Epidemiology. Foot-and-mouth disease under control in the UK Nature 2001 411 258 259 10.1038/35077149 11357118

[B28] Taubenberger JK Morens DM 1918 Influenza: the mother of all pandemics Emerg Infect Dis 2006 12 15 22 16494711 10.3201/eid1201.050979 PMC3291398

[B29] Ferguson NM Cummings DA Fraser C Cajka JC Cooley PC Burke DS Strategies for mitigating an influenza pandemic Nature 2006 442 448 452 10.1038/nature04795 16642006 PMC7095311

[B30] Pandemic (H1N1) 2009 in England: an overview of initial epidemiological findings and implications for the second wave http://www.hpa.org.uk/web/HPAwebFile/HPAweb_C/1258560552857

[B31] Fraser C Donnelly CA Cauchemez S Hanage WP Van Kerkhove MD Hollingsworth TD Griffin J Baggaley RF Jenkins HE Lyons EJ Jombart T Hinsley WR Grassly NC Balloux F Ghani AC Ferguson NM Rambaut A Pybus OG Lopez-Gatell H Alpuche-Aranda CM Chapela IB Zavala EP Guevara DM Checchi F Garcia E Hugonnet S Roth C Pandemic potential of a strain of influenza A (H1N1): early findings Science 2009 324 1557 1561 10.1126/science.1176062 19433588 PMC3735127

[B32] Goddard NL Kyncl J Watson JM Appropriateness of thresholds currently used to describe influenza activity in England Commun Dis Public Health 2003 6 238 245 14708275

[B33] Elliot A Syndromic surveillance: the next phase of public health monitoring during the H1N1 influenza pandemic? Euro Surveill 2009 14 1 3 19941780

[B34] Garske T Legrand J Donnelly CA Ward H Cauchemez S Fraser C Ferguson NM Ghani AC Assessing the severity of the novel influenza A/H1N1 pandemic BMJ 2009 339 b2840 10.1136/bmj.b2840 19602714

[B35] Espino JU Hogan WR Wagner MM Telephone triage: a timely data source for surveillance of influenza-like diseases AMIA Annu Symp Proc 2003 215 219 14728165 PMC1480215

[B36] Harcourt SE Smith GE Hollyoak V Joseph CA Chaloner R Rehman Y Warburton F Ejidokun OO Watson JM Griffiths RK Can calls to NHS Direct be used for syndromic surveillance? Commun Dis Public Health 2001 4 178 182 11732356

[B37] Doroshenko A Cooper D Smith G Gerard E Chinemana F Verlander N Nicoll A Evaluation of syndromic surveillance based on National Health Service Direct derived data--England and Wales MMWR Morb Mortal Wkly Rep 2005 54 Suppl 117 122 16177702

[B38] Smith GE Cooper DL Loveridge P Chinemana F Gerard E Verlander N A national syndromic surveillance system for England and Wales using calls to a telephone helpline Euro Surveill 2006 11 220 224 17370968

[B39] Edge VL Pollari F Ng LK Michel P McEwen SA Wilson JB Jerrett M Sockett PN Martin SW Syndromic Surveillance of Norovirus using Over-the-counter Sales of Medications Related to Gastrointestinal Illness Can J Infect Dis Med Microbiol 2006 17 235 241 18382634 10.1155/2006/958191 PMC2095074

[B40] Goldenberg A Shmueli G Caruana RA Fienberg SE Early statistical detection of anthrax outbreaks by tracking over-the-counter medication sales Proc Natl Acad Sci USA 2002 99 5237 5240 10.1073/pnas.042117499 11959973 PMC122753

[B41] Ginsberg J Mohebbi MH Patel RS Brammer L Smolinski MS Brilliant L Detecting influenza epidemics using search engine query data Nature 2009 457 1012 1014 10.1038/nature07634 19020500

