# Supplementary material for: Rapid detection of pandemic influenza in the presence of seasonal influenza
Source: BMC Public Health. 2010 Nov 24;10:726. doi: 10.1186/1471-2458-10-726 (PMC3001734; doi:10.1186/1471-2458-10-726)
Supplement: Additional file 1 — Details of the sampling of reported pandemic ILI cases. This details how the simulated pandemic cases were sampled at the GP level from the model-generated daily influenza infections at the postcode district level. [file 1471-2458-10-726-S1.DOC]

# Details of the sampling of reported pandemic ILI cases

The pandemic model generates, on a daily basis, the number of new influenza infections at the postcode district level. We convert these daily new infections into reported pandemic ILI cases at the health board level on a weekly basis. This is done as follows. First, we aggregate the model’s daily infections at the postcode area[[1]](#footnote-2) level by summing daily infections across all postcode districts belonging to a postcode area. From these aggregated daily infections, we then obtain the daily infections at the sentinel GP level in the proportion of the number of persons registered with the GP to the population of the postcode area to which the given sentinel GP belongs. (Here the two-step sampling process for getting the GP-level daily infections is necessary because it is often the case that the catchment area of a GP covers more than one postcode district.) Having obtained the GP-level daily infections, we then sample the daily reported pandemic ILI cases at different values of the case reporting rate α.

1. For example in the postcode ‘EH55 1XX,’ the ‘EH’ represents the postcode area while EH55 the postcode district. [↑](#footnote-ref-2)
