# Supplementary material for: Rapid detection of pandemic influenza in the presence of seasonal influenza
Source: BMC Public Health. 2010 Nov 24;10:726. doi: 10.1186/1471-2458-10-726 (PMC3001734; doi:10.1186/1471-2458-10-726)
Supplement: Additional file 2 — Smoothing of seasonal ILI data. This figure shows the outputs of the Poisson model for all the six influenza seasons from 2001-02 to 2006-07. [file 1471-2458-10-726-S2.DOC]

# Smoothing of seasonal ILI data

**Figure:** Smoothing of the joint probability distribution shown in Fig. 2 was done by simulating the Poisson model of the historical SERVIS ILI data discussed in the paper. The green *pluses* show the average value over *N* (=100) simulated data points shown by the blue *crosses*. Note we used N=10000 for calculating the joint probability distribution shown in Fig. 2. The observed weekly data are shown by *circles*.
