# Supplementary material for: Rapid detection of pandemic influenza in the presence of seasonal influenza
Source: BMC Public Health. 2010 Nov 24;10:726. doi: 10.1186/1471-2458-10-726 (PMC3001734; doi:10.1186/1471-2458-10-726)
Supplement: Additional file 3 — Detection thresholds for different (d, k) pairs and the selection of the best of the Mov-Avg Cusum (d, k) method. This describes the finding of the detection threshold values of different Mov-Avg Cusum models and the selection of the best among them to make a comparison with the WCR method. [file 1471-2458-10-726-S3.DOC]

# Detection thresholds for different (*d*, *k*) pairs and the selection of the best of the Mov-Avg Cusum (*d, k*) method

The Cusum thresholds, corresponding to all eight combinatorial pairs of (*d*, *k*), were determined by scanning different ranges of the values of the *d*-week upper weekly $Cusum_{t}^{+}$. (See the definition in the main paper.) First, for a given pair of (*d*, *k*), we calculate the weekly $Cusum_{t}^{+}$ values starting on week *t* (>*d*+7) from the seasonal ILI data of all 6 seasons (2001-02 to 2006-07). In the second step, we scan 6 series of the weekly $Cusum_{t}^{+}$ values against a probable detection threshold (we select a range of values starting from 0 and incrementing it by 0.05) to count the number of alarms in each season’s ILI data. An alarm is raised when the $Cusum_{t}^{+}$ value on a week is above the chosen probable threshold. Once we have the total counts of the false alarms from all 6 seasons, we calculate the specificity (defined in the main text) at this value. The process is repeated for the next value in the range. A detection threshold corresponding to a pre-specified specificity (*Sp*) for the given (*d*, *k*) is that value in the range for which the calculated specificity value crosses the pre-specified specificity *Sp* for the first time. Figures 1a and 1b illustrate the threshold values for *Sp*=99% and 95%.

**Figure 1a:** The Cusum thresholds for the specificity of 99% are marked by the vertical line on the x-axis. The plots are indicated by the (*d*, *k*) values.

**Figure 1b:** The Cusum thresholds for the specificity of 95% are marked by the vertical line on the x-axis. Again, the plots are indicated by the (*d*, *k*) values.

### Selection of the best Cusum (*d*, *k*) model

**Table:** The performances of the Cusum (*d*, *k*) models were compared in terms of cumulative detection (%) within the first N (=4, 5, …, 12) weeks of pandemic starting, averaged over 1800 *times* 33 runs. The (0, 1) model was selected for comparing the detection efficiency of the Mov-Avg Cusum method to that of our method.
