# Supplementary material for: Rapid detection of pandemic influenza in the presence of seasonal influenza
Source: BMC Public Health. 2010 Nov 24;10:726. doi: 10.1186/1471-2458-10-726 (PMC3001734; doi:10.1186/1471-2458-10-726)
Supplement: Additional file 4 — Seasonal SERVIS ILI data for the influenza season 2008-09. This figure presents the weekly time series of seasonal SERVIS ILI cases at the HB level for the season 2008-09. [file 1471-2458-10-726-S4.DOC]

# Seasonal SERVIS ILI data for the influenza season 2008-09

**Figure:** The SERVIS weekly ILI time series are shown by HBs for the season 2008-09 that include the 2009 influenza A(H1N1)v cases. Only 9 out the 13 participating health boards had one or more sentinel GPs that were reporting seasonal ILI cases.
